# Intercondylar notch volume in patients with posterior cruciate ligament tears and tibial avulsion injuries: a study applying computed tomography

**DOI:** 10.1186/s13018-022-03451-4

**Published:** 2022-12-23

**Authors:** Wen-Tao Huang, Kai Kang, Jin-Yu Yang, Hui Sun, Tong Li, Han Wang, Shi-Jun Gao

**Affiliations:** 1 Department of Orthopaedic Surgery, The Third Hospital of Hebei Medical University, Shijiazhuang, Hebei 050051 China; 2 Department of Pediatrics, Rongcheng People’s Hospital, Weihai, Shandong 264300 China; 3 Department of Ophthalmology, Shidao People’s Hospital Of Rongcheng, Weihai, Shandong 264308 China

**Keywords:** Posterior cruciate ligament, Femoral intercondylar notch, Volume, Body mass index

## Abstract

**Background:**

Two relatively common forms of injury exist in the posterior cruciate ligament (PCL) after the onset of trauma: PCL tear and tibial avulsion fracture. The mechanism for the occurrence of these different forms of injury is not known. Herein, we aimed to investigate this mechanism by comparing the intercondylar notch parameters between patients with PCL tears and those with PCL avulsion fractures of the tibial insertion.

**Methods:**

Fifty-three patients with PCL tears (37 male, 16 female: median age of 37 years: range 18–54 years) and 46 patients with avulsion fractures of tibial insertion (33 male, 13 female: median age of 33 years: range 18–55 years) were included in this study. Three-dimensional computed tomography (CT) was applied to measure the intercondylar notch width index and intercondylar notch volume. The intercondylar notch volume was simulated as the truncated-pyramid shape. Measurements of the top and bottom areas of this model were conducted on the slice containing the most proximal (*S*_1_) and most distal (*S*_2_) levels of Blumensaat’s line. Femoral condyle height (*h*) was defined as the vertical distance between two parallel planes, and the volume was calculated as *h*(*S*_1_ + *S*_2_ + √(*S*_1_*S*_2_))/3. The values of *S*_1_, *S*_2_, *h*, notch volume, the body mass index (BMI), intercondylar notch width (NW), femoral condylar width (FW) and notch width index (NWI) were compared among the PCL tear and avulsion-fracture groups.

**Results:**

The results show a significant difference in the *S*_2_ and normalized intercondylar notch volumes among patients with PCL tears and tibial avulsion injuries. Patients with PCL tears have smaller *S*_2_ and intercondylar notch volumes than those with tibial avulsion. There were no significant differences between the two groups in *S*_1_ or the 2D notch measurement parameters, such as the NW, FW and NWI. In addition, logistic regression analysis revealed notch volume and body mass index (BMI) as two significant independent predictors for PCL tears.

**Conclusion:**

Decreased intercondylar notch volume and increased BMI are associated with an increased incidence of PCL tears. The occurrence of PCL tears and tibial avulsion injuries is influenced by the femoral intercondylar notch volume, and the measurement of the notch volume could be useful for identifying patients at risk for PCL tears.

## Introduction

The exact incidence of posterior cruciate ligament (PCL) lesions requires further study and has been reported to range from 1 to 40% of all knee injuries [1, 2]. The PCL is the main restraining structure for posterior tibial translation, and the absence of the PCL, like the absence of the anterior cruciate ligament (ACL), may lead to subsequent meniscal and cartilage injuries and a rapid development of osteoarthritis, especially in young and active patients [3–6].

Particular knee morphologic risk factors have been reported to correlate with ACL and PCL injuries [7, 8]. Morphologic studies conducted on PCL injuries are limited, and among these potential risk factors is the controversial intercondylar femoral notch [9, 10]. By applying standard models of the knee established in Rosenberg and anteroposterior radiographic views, van Kuijk et al. found that a smaller and more sharply angled intercondylar notch is associated with PCL injury [9]. In contrast, the results of an MRI study, which was conducted by Liu Fei et al., suggested that a stenotic intercondylar notch is not a risk factor for PCL injury [10].

In the past, researchers have regularly applied two-dimensional (2D) indicators such as the notch width index (NWI) to describe the morphology of intercondylar notches [11]. However, the intercondylar notch is a complex three-dimensional (3D) space, and the 2D notch measurement parameters may be plane and location specific [12]. Therefore, the application of 2D parameters to characterize the 3D structure of the intercondylar notch may be inadequate. Several studies have shown that the intercondylar notch volume, as a 3D parameter, is a superior indicator [12–14].

Measurement of the volume of the intercondylar notch is a complicated operational project. Iriuchishima et al. proposed a method to simulate the volume of the intercondylar notch with a truncated-pyramid shape, which is a creative idea [15, 16]. With this approach to measurement, they investigated intercondylar notches with a different morphology of Blumensaat's line and patients with and without ACL tears [15, 16]. In fact, these efforts demonstrate the reliability of their method.

As the traumatic process experienced by patients with tibial avulsion fractures is nearly identical to that of patients with PCL tears [17], patients with PCL tibial avulsion fractures were selected as the control group for this study. The aim of the present study was to use thin-layer computed tomography (CT) to compare the intercondylar notch parameters between patients with PCL tears and those with PCL avulsion fractures of the tibial insertion. The authors hypothesize that there would be a significant difference between patients with PCL tears and those with tibial avulsion injuries.

## Materials and methods

The requirement to obtain informed consent from participants was waived due to the retrospective nature of the study. The study was approved by the Ethical Review Committee of the Third Hospital Hebei Medical University.

Fifty-three patients who were treated surgically for primary PCL tears (37 male, 16 female: median age of 37 years: range 18–54 years) and 46 patients with avulsion fractures of the tibial insertion (33 male, 13 female: median age of 33 years: range 18–55 years) at our hospital were included in this study. The exclusion criteria were multiple ligament injuries, previous surgery on the affected knee, osteoarthritis Outerbridge scale ≥ grade 3, revision PCL surgery and significant osteoporosis diagnosis. The demographics of the patients are shown in Table 1.

**Table 1:** Baseline clinical characteristics among the PCL tear and avulsion fracture group

	PCL tear (53)	Avulsion fracture (46)	*P* value
Age, years^b^	36.66 ± 10.62	34.04 ± 10.13	0.22
Female/Male^c^	16/37	13/33	0.83
Height^b^	171.15 ± 8.86	170.35 ± 7.70	0.63
Weight^b^	78.92 ± 13.49	72.89 ± 17.72	0.06
BMI^b^	26.87 ± 3.48	25.00 ± 5.08	0.03^a^

Data are presented as the mean ± standard deviation (median ± interquartile range)

PCL, posterior cruciate ligament; BMI, body mass index

^a^Significant difference was found

^b^Student’s *t* test

^c^Chi-square test

### Computed tomography measurement

All thin-section CT scans of participants were performed with a 64-slice CT scanner (Somatom Sensation 64, Siemens, Erlangen, Germany). Mimics Research (Version 21.0.0.406) software was used to perform the 3D reconstruction, position adjustment, reslicing and measurement of the images. The image position on the sagittal view of the 3D-CT was adjusted to match the shape of the medial and lateral femoral condyles to the maximal extent. Measurements of the indicators were performed by two board-certified arthroscopic surgeons using a blinded method. The observers performed the measurements two times each, and the average was used as the data.

**Fig. 1:**
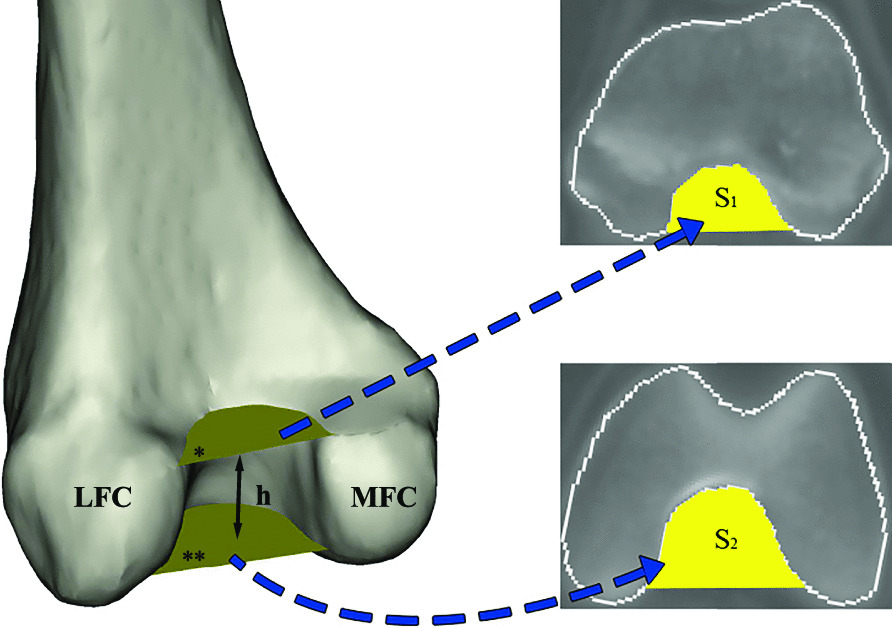
Axial femoral intercondylar notch area measurement in the posterior view of a right femoral condyle. Axial femoral intercondylar notch areas were measured at the most proximal and most distal height of Blumensaat’s line. When calculating the volume of the truncated pyramid shape, the proximal (*) and distal area (**) were regarded as the upper and lower base (*S*_1_ and *S*_2_) and the height (*h*) of the truncated pyramidal shape was the height of the intercondylar notch

The intercondylar notch volume was measured according to the method described by Iriuchishima et al. [15, 16]. In brief, after determining the long axis of the sagittal femoral bone, axial femoral intercondylar notch areas (top and bottom surface area of the truncated pyramidal) were measured at the height of the most proximal (*S*_1_) and most distal end (*S*_2_) of Blumensaat’s line. The posterior edge of the intercondylar notch is defined as the point where the slope changes abruptly on the medial and lateral femoral condyles. By outlining the intercondylar notch and connecting the posterior edge of the intercondylar notch with a straight line, the axial notch area was measured. The height of the truncated pyramidal shape was the height of the intercondylar notch. The volume was calculated as volume (mm^3^) = *h*(*S*_1_ + *S*_2_ +√(*S*_1_*S*_2_))/3 insert (Fig. 1).

**Fig. 2:**
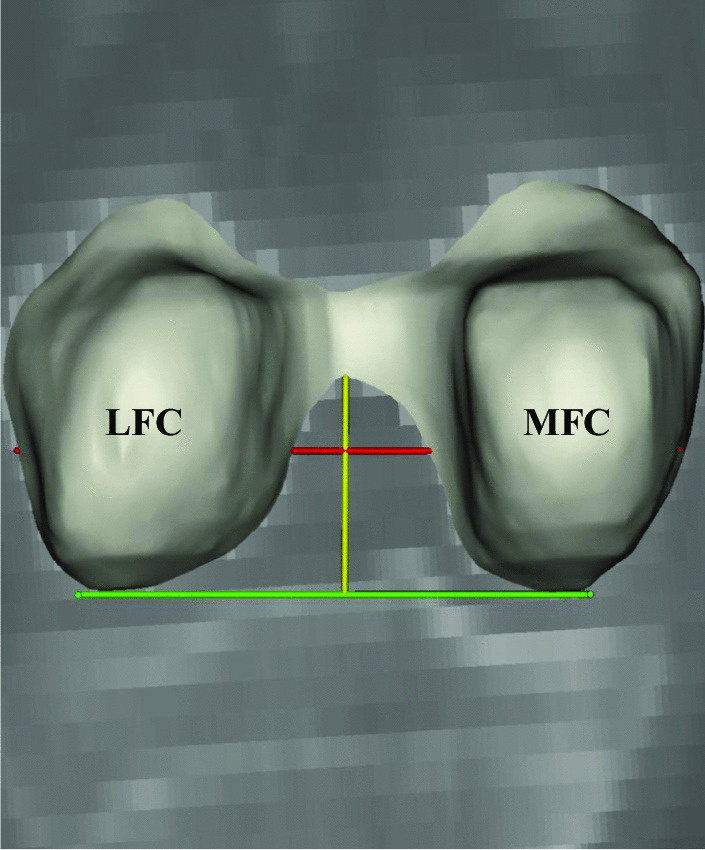
Measurement of the intercondylar notch in the posterior view of a right femoral condyle. The green line represents the tangent line of the distal end of the femoral condyle. The yellow line represents the intercondylar depth. The red line represents the intercondylar notch width (NW)

The NWI was obtained by dividing the notch width (NW) by the femoral condyle width (FW). This measurement was based on the method described by Stein et al. and Alentorn et al. [18, 19]. On the coronal plane showing the popliteal groove, a tangent to the distal end of the femoral condyles and a line vertical to this tangent were established. The intercondylar depth was defined as the vertical distance from the top of the intercondylar notch to the tangent line. At the upper third of the intercondylar depth, parallel to the tangent line, the NW and FW were measured (Fig. 2).

### Statistical analysis

Statistical analyses were performed using SPSS (IBM Corp., Armonk, NY), version 26, and the *P* value for statistical significance was set at < 0.05. *S*_1_, *S*_2_, *h*, notch volume, NW, FW, NWI and notch volume/body height were compared between the PCL tear and tibial avulsion groups. The results of inter- and intraobserver reliability were assessed using the intraclass correlation coefficient (ICC).

The Shapiro‒Wilk test and the Levene test were applied for data normality and homogeneity of variance tests. Student’s *t* test was applied to variables (*S*_1_, *S*_2_, *h*, notch volume and FW) that met the two conditions, and nonparametric variables (NW, NWI and notch volume/body height) were compared using the Mann‒Whitney *U* test (Table 2). Binary logistic regression was applied to determine the significant independent predictors of ligament tears.

**Table 2:** Comparison of the morphological characteristics of the PCL tear and avulsion fracture groups

	PCL tear (53)	Avulsion fracture (46)	*P* value
*S*_1_^b^	189.89 ± 45.86 mm^2^	201.46 ± 40.69 mm^2^	0.19
*S*_2_^b^	287.93 ± 75.22 mm^2^	339.08 ± 70.78 mm^2^	0.001^a^
h^b^	25.16 ± 2.59 mm	24.68 ± 2.44 mm	0.35
V^b^	6.48 ± 2.37 cm^3^	7.39 ± 2.17 cm^3^	0.051
NW^c^	15.41 ± 2.81 mm	15.82 ± 2.62 mm	0.45
FW^b^	78.33 ± 5.84 mm	78.79 ± 6.18 mm	0.70
NWI^c^	0.20 ± 0.04	0.20 ± 0.03	0.46

Data are presented as the mean ± standard deviation (median ± interquartile range)

PCL, posterior cruciate ligament; *S*_1_, intercondylar notch area of the slice containing the most proximal level of Blumensaat’s line; *S*_2_, intercondylar notch area of the slice containing the most distal level of Blumensaat’s line; h, height of the intercondylar notch; V, intercondylar notch volume; NW, intercondylar notch width; FW, femoral condylar width; NWI, notch width index

^a^A significant difference was found

^b^Student’s *t* test

^c^Mann‒Whitney *U* test

## Results

### Patient demographics

No significant difference was determined between the two groups in terms of baseline characteristics, such as age and sex. The same was observed in terms of height and weight. However, the body mass index (BMI) in the PCL tear group was significantly higher than that in the tibial avulsion group (Table 1).

### Computed tomography measurement

There were no significant differences between the two groups in the two-dimensional (2D) notch measurement parameters, such as NW, FW and NWI. *S*_2_ in the PCL tear group (287.93 ± 75.22 mm^2^, *P* = 0.001) was significantly smaller than that in the tibial avulsion group (339.08 ± 70.78 mm^2^). However, no significant difference was found in *S*_1_ and *h* between the two groups.

**Fig. 3:**
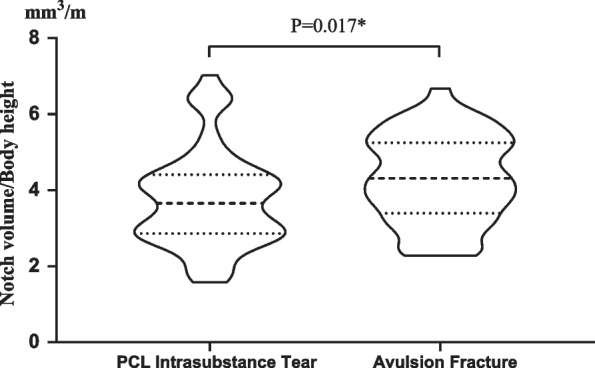
Violin plot demonstrated the differences in the parameter notch volume/body height of the two groups

Although the results of this study could not be considered significantly different with respect to volume comparisons (*P* = 0.051), after normalization by body height, the results suggested significant differences (Table 2 and Fig. 3). In addition, the logistic regression analysis revealed notch volume (OR = − 0.21, *P* = 0.03) and BMI (OR = 0.12, *P* = 0.022) as two significant independent predictors for ligament tears (Table 3).

**Table 3:** Odds ratio and *P* value of the logistic regression analysis for predictor variables

	OR	*P* value
V	− 0.21	0.03^a^
BMI	0.12	0.022^a^

OR, odds ratio; V, intercondylar notch volume; BMI, body mass index.

^a^Significant difference was found

Intra- and interobserver reliability are shown in Table 4.

**Table 4:** Results expressed as the ICC (95% CI) of the inter- and intraobserver reliability analysis

	Interobserver reliability	Intraobserver reliability
*S*_1_	0.872 (0.835, 0.902)	0.898 (0.865, 0.932)
*S*_2_	0.899 (0.868, 0.922)	0.921 (0.896, 0.941)
h	0.904 (0.875, 0.927)	0.875 (0.668, 0.938)
NW	0.906 (0.878, 0.982)	0.923 (0.898, 0.942)
FW	0.914 (0.887, 0.934)	0.945 (0.927, 0.958)

ICC, intraclass correlation coefficient; CI, confidence interval; *S*_1_, intercondylar notch area of the slice containing the most proximal level of Blumensaat’s line; *S*_2_, intercondylar notch area of the slice containing the most distal level of Blumensaat’s line; h, femoral condyle height; NW, intercondylar notch width; FW, femoral condylar width

## Discussion

The most important finding of the present study was that decreased notch volume and increased BMI were risk factors for PCL tears during injuries. After normalizing the results with body height, the indicator notch volume/body height was significantly smaller among patients with PCL tears than among those with avulsion fractures. Furthermore, the area of the bottom (distal portion) of the truncated pyramid was significantly smaller in patients with PCL tears than in patients with avulsion fractures, which may account for the difference in results.

An X-ray study conducted by Mininder et al. concluded that the NWI was significantly smaller in children and adolescents with ACL tears than in patients with avulsion fractures [20]. The NWI is a widely accepted and recognized 2D indicator for measuring the intercondylar notch with high sensitivity [14, 21]. However, according to the results of this study, there was no significant difference in NWIs between patients with PCL tears and those with avulsion fractures. It is possible that for patients with PCL injury, the NWI does not describe the intercondylar notch as sufficiently as it does ACL. To our knowledge, this is the first study to assess the morphological differences between PCL tears and avulsion fractures. This result is all the more convincing because the patients have experienced an extremely similar mechanism of injury [17].

Intercondylar notch volume, as a 3D indicator, has been supported by some published studies to describe intercondylar notch morphology precisely [12, 15]. However, to calculate notch volume, the clinician may have to measure the notch area from all slices, which implies a large workload [13, 15, 16]. This makes it unworkable for the daily clinical routine. The simulation of the intercondylar notch volume as a truncated-pyramid shape proposed by Iriuchishima et al. largely changed this situation [15, 16]. Although the accurate notch volume was not measured directly, the significant advantage of this methodology was that the volume measurement could be simulated using the notch height and the upper and lower base areas only. Their subsequent research on the effect of Blumensaat's line on the volume of the intercondylar notch also showed that this is a reliable simulation method [16].

It is known that there is a positive correlation between the volumes of the intercondylar notch, PCL and ACL [14]. The results of this study indicated that when injury occurs, individuals with smaller ligament volumes were more likely to develop PCL tears, while individuals with larger PCL volumes tended to develop avulsion fractures. Based on these findings, we hypothesize that the smaller PCLs are injured because of the smaller force they can withhold, while avulsion fractures occur in patients with larger ligaments because the force that the PCLs can withhold is greater than the strength of the tibial attachment area.

The injury mechanism of PCL tears and tibial avulsion is remarkably similar [17], and in many cases, the injury is the result of a posteriorly directed force acting on the proximal tibia [22]. The difference, however, is that tibial avulsion is mostly caused by two-wheeler accidents, a form of injury that is rare in the Western world [1, 2, 23], which means that there are subtle discrepancies in the external forces applied at the time of trauma. Limited studies have been conducted on PCL tibial avulsion, and the specific causes of the injury still need to be further investigated. Significant differences in the indicator of BMI may influence the forces exerted on the PCL. This appears to be consistent with previous studies showing that slower loading rates could preferentially result in tibial avulsion fracture [20, 24]. However, based on current knowledge, it seems difficult to explain the influence of BMI in the process of injury.

A further understanding of anatomical variants is the basis for screening individuals susceptible to PCL injury and individualized ligament surgery [7, 8, 25]. A meta-analysis presented by Yulun et al. reported that ACL injury prevention programs can significantly reduce injury rates [26]. There appear to be no prevention programs for PCL injury, but only as basic research progresses will these programs emerge [14]. The volume of the intercondylar notch can be modified intraoperatively by intercondylar notchplasty [27, 28]; however, the surgeon cannot change the size of the ligament. It is not clear whether this intervention can prevent PCL injury or reduce the rate of postoperative reinjury, and future studies are still needed.

The principal limitations of this study were as follows: (1) the total number of patients involved in this study was only 99 individuals. Due to the low incidence of tibial avulsion fractures, no matching for sex and age was performed with the PCL tear group. Owing to anatomical variability, studies with larger sample sizes are needed. (2) The study was retrospective in nature and only evaluated patients with PCL lesions in Hebei Province, China. Studies that take the variability across regions and ethnicities into account are needed.

## Conclusion

Decreased intercondylar notch volume and increased BMI were associated with an increased incidence of PCL tears. The occurrence of PCL tears and tibial avulsion injuries is influenced by the femoral intercondylar notch volume, and the measurement of the notch volume can be useful for identifying patients at risk for PCL tears. Clinical Relevance: Identification of morphological risk factors can help to reduce injuries in individuals at high risk for PCL ruptures or even to reduce the rate of recurrence after PCL reconstruction.

## Data Availability

The data of this work are available on request to the corresponding author.

## Abbreviations

PCL: Posterior cruciate ligament
ACL: Anterior cruciate ligament
LFC: Lateral femoral condyle
MFC: Medial femoral condyle
3D: Three-dimensional
2D: Two-dimensional
CT: Computed tomography
*S*_1_: Intercondylar notch area of the slice containing the most proximal level of Blumensaat’s line
*S*_2_: Intercondylar notch area of the slice containing the most distal level of Blumensaat’s line
*h*: Femoral condyle height
NW: Intercondylar notch width
FW: Femoral condylar width
NWI: Notch width index
BMI: Body mass index
V: Intercondylar notch volume
ICC: Intraclass correlation coefficient
CI: Confidence interval
OR: Odds ratio
